# Factors influencing the perception of illness in patients with Parkinson’s disease

**DOI:** 10.3389/fneur.2026.1773273

**Published:** 2026-04-28

**Authors:** Wen Ma, Yafeng Cui, Yuqing Li, Min Zhou, Xia Wang, Le Xu

**Affiliations:** The First Affiliated Hospital of Anhui Medical University, Hefei, Anhui, China

**Keywords:** cross-sectional study, illness perception, influencing factors, nursing care, Parkinson’s disease

## Abstract

**Objective:**

To investigate the status and factors influencing the perception of illness in patients with Parkinson’s disease to provide a reference for the formulation of targeted intervention measures.

**Methods:**

The study used a cross-sectional design. Convenience sampling was used to enroll 128 patients diagnosed with Parkinson’s disease in the Neurology Department of a tertiary hospital in Anhui Province, China, between February and November 2023. The investigation was conducted using a general information questionnaire, and the Chinese version of the Revised Illness Perception Questionnaire (CIPQ-R). Data were analyzed using independent samples t-tests, one-way analysis of variance, and multiple linear regression in SPSS 23.0.

**Results:**

Significant levels of negative perception were identified, with high scores for emotional representation 21.14 ± 3.44 and consequence 19.45 ± 3.03. Multiple linear regression analysis showed that gender, marriage, education, age, disease duration, H-Y stage influenced illness perception in the patients (*p <* 0.05).

**Conclusion:**

Patients with Parkinson’s disease generally have a negative perception of the disease, particularly in terms of consequences and emotional representation. Women, widows, patients with low levels of education, and those with prolonged disease duration were at high risk of negative perception. These findings provide empirical evidence for the clinical identification of high-risk groups and the formulation of targeted psychological interventions, such as strengthening social support and training in coping skills.

## 1 Introduction

Parkinson’s disease (PD) is the most common neurodegenerative disease in the world after Alzheimer’s disease (1). Data from the Global Burden of Disease Study indicate that PD prevalence is increasing significantly, with an increase in the number of PD cases from 7 million in 2015 to approximately 13 million in 2024 (2). In China, it is estimated that with population aging and increasing life expectancy, the number of individuals with PD will reach 5 million by 2030 (3, 4). Neurological disorders are a leading cause of global disability, and PD ranks among the fastest-growing contributors in terms of disability burden (5). The disease is characterized by motor symptoms, including bradykinesia, muscle rigidity, and resting tremor, as well as a range of non-motor symptoms, such as cognitive decline, autonomic dysfunction, sleep disturbances, and anxiety or depression. The symptom profile worsens progressively with disease duration and advancing age, resulting in impairment of multiple functions and significant reductions in patients’ health-related quality of life (6). PD not only imposes substantial economic burdens on families and society but also increases the need for healthcare personnel and long-term care resources, thereby further straining global health systems (7).

Illness perception, derived from the Common-Sense Model of Self-Regulation, refers to patients’ cognitive representations and understanding of their illness or specific symptoms (8). It involves the process by which individuals suffering from a disease or perceiving a threat to their health, interpret, analyze, and make sense of their current symptoms or condition based on pre-existing illness-related knowledge and personal experience (9). Research evidence indicates that illness perception is a key determinant of patient behavior and can significantly influence the onset, progression, and prognosis of disease (10). Therefore, an understanding of illness perception in patients with PD is necessary for the development of effective treatment strategies by healthcare professionals.

In recent years, illness perception, a key factor that influences the psychological adaptation and clinical outcomes of patients, has attracted increasing attention in the field of neurodegeneration. Studies on amyotrophic lateral sclerosis (ALS) have demonstrated that a patient’s perception of illness is closely linked to their emotional state and quality of life (11). Research on Huntington’s disease (HD) has confirmed that perceptions of symptom attributions, treatment expectations, and disease variability are effective in predicting anxiety and depression levels in patients (12). A review of Alzheimer’s disease (AD) suggests that by identifying and challenging adverse illness perceptions and fostering alternative perceptions, illness perceptions can be modified, thereby improving psychosocial outcomes (13).

Notable progress has also been achieved in research on Parkinson’s disease (PD). Early studies verified that psychosocial factors, such as perceived control, are critically important in a patient’s adaptation to the disease (14). Subsequent research has shown that the patient’s perceptions of disease consequences, personal control capabilities, and emotional responses not only explain observed differences in anxiety and depressive symptoms (15), but also represent independent predictors of mental health and quality of life (16). Recent studies have focused on the dynamic characteristics of illness perception, indicating that “personal control” can be changed through psychological intervention (17). Qualitative research has also shown that acceptance and denial of the disease often intertwine and coexist in complex ways to jointly shape the trajectories of disease adaptation in patients (14).

While the studies described above have laid a solid foundation for understanding illness perception in patients with PD, most of the evidence has been derived from Western populations. A patient’s perception of illness is profoundly influenced by their individual experiences, social support, and cultural context (18). In China, the influence of traditional family values, prevailing social support models, and the unique characteristics of the healthcare system may result in distinct cultural specificities in disease perception by patients with PD. However, there have been few systematic investigations of this issue in Chinese patients with PD.

To address this gap, the present study aimed to investigate the current status of illness perception and its influencing factors among Chinese patients with PD. The findings are expected to provide a foundation for the development of culturally tailored intervention strategies, facilitate the early identification of high-risk groups, promote psychological adaptation by ameliorating negative illness perceptions, and ultimately enhance the quality of life of patients.

## 2 Methods

### 2.1 Participants

The study participants were recruited from the inpatient neurology ward of the First Affiliated Hospital of Anhui Medical University between February and November 2023 using convenience sampling. The diagnosis of PD was confirmed for each potential participant by the attending physician. Following this, members of the research team then approached eligible inpatients. After providing a detailed explanation of the purpose and procedures of the study, interested patients were screened against the predetermined inclusion and exclusion criteria. All patients who met these criteria and provided written informed consent were enrolled while they were hospitalized. Baseline assessments and questionnaire administration were conducted in the inpatient setting when the clinical condition of the patients was considered stable by the treating medical team.

### 2.2 Inclusion criteria

The inclusion criteria were as follows: (1) confirmation of diagnosis based on the revised diagnostic criteria for PD of the International Parkinson and Movement Disorder Society (19); (2) ability to communicate both verbally and in writing; (3) provision of informed consent; and (4) voluntary participation in the study.

### 2.3 Exclusion criteria

The exclusion criteria were: (1) the presence of major psychiatric disorders (e.g., schizophrenia, bipolar disorder, major depressive disorder with psychotic features, or dementia) that could impair cognitive assessment or communication; (2) severe visual or auditory impairments; (3) difficulties in communication; (4) concurrent malignancies; and (5) significant organ dysfunction involving the heart, brain, or kidneys. It should be noted that the criterion of significant organ dysfunction was included primarily for participant safety and to reduce the potential confounding effects of severe non-PD related comorbidities on the study outcomes.

A total of 142 questionnaires were distributed, of which 128 were completed and deemed valid, resulting in a valid response rate of 90.14%.

### 2.4 Measurements

#### 2.4.1 General information questionnaire

The questionnaire was developed by the researchers based on a review of relevant literature and in alignment with the objectives and content of the study (20). The questionnaire collected data on demographic and clinical variables such as gender, age, marital status, educational level, disease duration, type of medical expense coverage, and Hoehn-Yahr stage.

#### 2.4.2 The Chinese illness perception questionnaire-revised (CIPQ-R)

The CIPQ-R has been validated in Chinese chronic disease populations (20). The questionnaire comprises three sections with 70 items in total. The first section assesses illness identity using 14 common symptoms. The second section contains 38 items measuring seven dimensions of illness perception: timeline (acute/chronic), consequences, personal control, treatment control, illness coherence, timeline cyclical, and emotional representation. The third section (18 items), which explores patients’ beliefs about the causes of their illness (e.g., heredity, stress, lifestyle), was not included in the present analysis. Cronbach’s alpha coefficients for the seven dimensions in this study ranged from 0.66 to 0.87, indicating acceptable internal consistency.

### 2.5 Data collection

Paper-based questionnaires were used for data collection. Before the formal survey, the investigators received standardized training that included a detailed review of the questionnaire contents and instructions on data collection. Written permission was obtained from the relevant clinical departments of the hospital. During data collection, a standardized script was used to inform participants about the purpose of the questionnaire and provide instructions for completion. If participants experienced difficulties in understanding, the investigators provided clarification using consistent, non-leading language. For participants who were unable to complete the questionnaire independently due to physical limitations (e.g., severe tremor, visual difficulties) or fatigue, trained research staff read the questions aloud and recorded the participants’ verbatim responses, ensuring that all answers reflected the participants’ own views. These procedures ensured the authenticity and accuracy of the collected data.

### 2.6 Ethical considerations

This study has been approved by the Ethics Committee of the First Affiliated Hospital of Anhui Medical University (ethics approval no. PJ 2023-05-16). All participants were informed of the purpose of the study and signed the informed consent form. This study adhered to the provisions of the Helsinki Declaration. To protect the privacy and human rights of the participants, this study adopted the method of anonymous data collection.

### 2.7 Statistical analysis

Data were entered using EpiData (version 3.1) (21) and analyzed with SPSS 23.0 (22). Univariate analyses, including independent-samples t-tests and one-way ANOVA, were first conducted to examine associations between demographic and clinical characteristics and the individual dimensions of illness perception. Variables with a *p*-value < 0.05 in the univariate analyses were included as candidate variables in subsequent multivariable models. Multiple linear regression with stepwise selection was then performed to identify independent predictors of each dimension of illness perception, with adjustments for potential confounders. The stepwise procedure employed entry and removal probabilities of 0.05 and 0.10, respectively. Multicollinearity was assessed using variance inflation factors (VIF), with VIF values < 5 indicating acceptable levels of collinearity. A two-tailed *p*-value < 0.05 was considered statistically significant.

## 3 Results

### 3.1 Demographic and clinical characteristics of patients with Parkinson’s disease

A total of 128 patients with PD were enrolled in the study, of whom 70 were male (54.7%) and 58 were female (45.3%). In terms of educational attainment, 70 patients (54.7%) had completed primary school or below, 48 (37.5%) had attended junior or senior high school, and 10 (7.8%) had received a college education or higher. In terms of disease severity, 64 patients (50%) were classified as being Hoehn and Yahr (H-Y) stages 1–2, while the remaining 64 patients (50%) were classified as stages 2.5–5. Further details are presented in Table 1.

**Table 1:** Demographic and clinical characteristics of patients with Parkinson’s disease.

Item	*n*	Percentage
Age		
≤60	60	46.9
≥61	68	53.1
Gender		
Male	70	54.7
Female	58	45.3
Marital status		
Married	124	96.9
Widowed or unmarried	4	3.1
Educational level		
Primary school and below	70	54.7
Junior high school or senior high school	48	37.5
College degree or above	10	7.8
Family monthly income		
≤1,000 RMB/month	56	43.8
1,001–3,000 RMB/month	44	34.4
>3,000 RMB/month	28	21.9
Type of health insurance		
medical insurance for urban employees	28	21.9
medical insurance for residents	12	9.4
New rural cooperative medical insurance scheme	88	68.8
Disease duration		
1–5 year	86	67.2
6–10 year	26	20.3
≥11 year	14	10.9
H-Y staging		
Grade 1–2	64	50
Grade 2.5–5	64	50

### 3.2 Status of illness perception in patients with Parkinson’s disease

Table 2 shows the illness perception score of patients with Parkinson’s disease and the score range and significance of each subscale. For example, in the Emotional Representations subscale (range 6–30), a score of 20 reflects moderate emotional distress, whereas a score of 10 would indicate minimal emotional impact; our mean score of 21.14 suggests that, on average, patients in our sample experience substantial emotional burden related to PD.

**Table 2:** Illness perception scores in patients with Parkinson’s disease.

Item	Score range	x̅ ± s	Meaning
Illness identity	0–14	3.89 ± 2.73	Higher scores indicate that patients attribute more symptoms to Parkinson’s disease.
Timeline (acute/chronic)	6–30	15.52 ± 1.19	Higher scores reflect a stronger belief that PD is a chronic, long-term condition.
Consequence	6–30	19.45 ± 3.03	Higher scores indicate greater perceived negative impact of PD on life.
Personal control	5–25	18.45 ± 2.23	Higher scores reflect stronger perceived ability to manage the illness.
Treatment control	5–25	19.91 ± 1.83	Higher scores indicate greater confidence in treatment effectiveness.
Illness coherence	5–25	17.17 ± 2.45	Higher scores reflect a clearer, more coherent understanding of PD.
Timeline cyclical	4–20	11.33 ± 2.31	Higher scores indicate a stronger belief that symptoms are cyclical and recurring.
Emotional representation	6–30	21.14 ± 3.44	Higher scores reflect more severe negative emotional distress related to PD.

### 3.3 Analysis and comparison of illness perception scores in relation to demographic characteristics

Table 3 presents the univariate comparisons of illness perception scores across different demographic and clinical subgroups. These exploratory analyses identified multiple factors associated with illness perception dimensions, which were subsequently entered into multivariable regression models to determine independent predictors. Statistically significant differences were observed in illness identity scores according to patient gender, educational level, medical payment method, disease duration, and H-Y stage (*p* < 0.05). Significant differences in disease chronicity scores were observed in terms of marital status, monthly income, and disease duration (*p* < 0.05). For the consequence subscale, significant differences were found between groups defined by gender and disease duration (*p* < 0.05). Personal control scores differed significantly in terms of both age and disease duration (*p* < 0.05), while the scores for illness coherence showed significant differences in relation to gender, marital status, and educational level (*p* < 0.05). Differences in timeline cyclical scores were significant in terms of educational level and disease duration (*p* < 0.05), and the scores for emotional representation varied markedly in terms of age, gender, and educational level (*p* < 0.05). Detailed results are presented in Table 3.

**Table 3:** Comparative analysis of illness perception scores in relation to different demographic characteristics.

Variable	Item	Subgroup	Mean ± SD	Test statistic	*p-*value
Age	Personal control	≤60 years	18.00 ± 2.59	*t* = −2.142	0.034
		≥61 years	18.85 ± 1.78		
	Emotional representation	≤60 years	21.90 ± 2.61	*t* = 2.45	0.016
		≥61 years	20.47 ± 3.93		
Gender	Illness identity	Male	3.37 ± 1.82	*t* = 5.786	0.018
		Female	4.52 ± 3.45		
	Consequence	Male	18.91 ± 3.15	*t* = 5.033	0.027
		Female	20.10 ± 2.77		
	Illness coherence	Male	16.74 ± 2.49	*t* = 4.882	0.029
		Female	17.69 ± 2.31		
	Emotional representation	Male	20.29 ± 3.82	*t* = 10.260	0.002
		Female	22.17 ± 2.58		
Marital status	Timeline (acute/chronic)	Married	15.45 ± 1.50	*t* = −0.357	<0.01
		Widowed/unmarried	17.50 ± 1.73		
	Illness coherence	Married	17.15 ± 2.49	*t* = −3.831	<0.01
		Widowed/unmarried	18.00 ± 0.00		
Educational level	Illness identity	Primary or below	4.37 ± 3.07	*F* = 5.55	0.005
		Junior/Senior high	3.54 ± 2.18		
		College or above	2.20 ± 1.69		
	Illness coherence	Primary or below	17.63 ± 2.42	*F* = 3.12	0.048
		Junior/Senior high	16.50 ± 2.44		
		College or above	17.20 ± 2.15		
	Timeline cyclical	Primary or below	11.71 ± 2.38	*F* = 5.62	0.005
		Junior/Senior high	11.04 ± 2.25		
		College or above	10.00 ± 2.31		
	Emotional representation	Primary or below	21.63 ± 2.86	*F* = 3.384	0.037
		Junior/Senior high	20.17 ± 4.17		
		College or above	22.40 ± 2.07		
Monthly income	Timeline (acute/chronic)	≤1,000 RMB	15.36 ± 1.05	*F* = 3.22	0.043
		1,001–3,000 RMB	15.86 ± 1.07		
		>3,000 RMB	15.29 ± 1.51		
Insurance type	Illness identity	Urban employee	2.86 ± 2.14	*F* = 6.594	0.002
		Resident	3.00 ± 0.85		
		New Rural Coop	4.34 ± 2.96		
Disease duration	Illness identity	1–5 years	4.16 ± 2.89	*F* = 4.29	0.016
		6–10 years	2.85 ± 2.59		
		≥11 years	4.57 ± 1.09		
	Timeline (acute/chronic)	1–5 years	15.40 ± 1.09	*F* = 8.42	<0.001
		6–10 years	15.92 ± 0.27		
		≥11 years	15.43 ± 2.34		
	Consequence	1–5 years	19.42 ± 3.10	*F* = 3.47	0.034
		6–10 years	18.69 ± 3.11		
		≥11 years	21.29 ± 1.73		
	Personal control	1–5 years	18.63 ± 2.09	*F* = 4.07	0.019
		6–10 years	18.62 ± 2.32		
		≥11 years	16.86 ± 2.51		
	Timeline cyclical	1–5 years	11.42 ± 2.40	*F* = 4.18	0.018
		6–10 years	10.54 ± 1.99		
		≥11 years	12.43 ± 1.99		
H-Y stage	Illness identity	Stage 1–2	3.34 ± 2.81	*t* = −2.30	0.023
		Stage 2.5–5	4.44 ± 2.56		

For variables with more than two groups, the F-test (ANOVA) was used; for two-group comparisons, the t-test was applied. *p*-values are based on the original analysis, with significance set at *p* < 0.05.

### 3.4 Multiple linear stepwise regression analysis of influencing factors of Parkinson’s illness perception

Table 4 presents the results of the multiple linear stepwise regression analysis of factors influencing illness perception in patients with Parkinson’s disease. The variable assignments were as follows: gender (Male = 1, Female = 2), age (≤60 years = 1, ≥61 years = 2), educational level (Primary school and below = 1, Junior high school or senior high school = 2, College degree or above = 3), H-Y stage (Grade1-2 = 1, Grade 2.5–5 = 2), and disease duration (1–5 years = 1, 6–10 years = 2, ≥11 years = 3). Marital status was not included in the regression equation due to uneven group distribution (124 married vs. 4 widowed or unmarried).

**Table 4:** Multiple linear stepwise regression analysis of influencing factors of Parkinson’s disease perception.

Illness perception factor	Associated factor	β	SE	Beta	t-value	VIF
Illness identity	Educational level	−1.23	0.35	−0.289	−3.519**	1.054
	Age	−1.765	0.451	−0.324	−3.917**	1.073
	H-Y staging	1.379	0.441	0.254	3.128**	1.028
	Gender	1.136	0.442	0.208	2.568*	1.030
Timeline (acute/chronic)	H-Y staging	0.428	0.211	0.179	2.031*	1.000
Consequence	Educational level	−1.043	0.418	−0.219	−2.496*	1.051
	Age	−1.191	0.533	−0.196	−2.236*	1.046
	Gender	1.123	0.529	0.184	2.125*	1.027
Personal control	Disease duration	−0.722	0.283	−0.221	−2.552*	1.008
	Age	0.904	0.387	0.202	2.333*	1.008
Illness coherence	Gender	0.984	0.434	0.200	2.268*	1.000
Timeline cyclical	Educational level	−0.814	0.317	−0.225	−2.568*	1.000
Emotional representation	Gender	2.136	0.582	0.309	3.668**	1.013
	Age	−1.722	0.581	−0.249	−2.962**	1.013

*p < 0.05, **p < 0.01.

The analysis revealed that educational level, age, gender, H-Y stage, and disease duration were the main factors influencing various dimensions of illness perception, with different factors associated with each dimension. All variance inflation factors (VIF) in the model were less than 2, indicating no significant multicollinearity among the independent variables.

Specifically, in the identity of illness dimension, older age (*β* = −0.324, *p <* 0.01), lower educational level (*β* = −0.289, *p <* 0.01), higher H-Y stage (*β* = 0.254, *p <* 0.01), and female gender (*β* = 0.208, *p <* 0.05) were associated with a stronger tendency to report perceived symptoms. In the timeline (acute/chronic) dimension, only H-Y stage (*β* = 0.179, *p <* 0.05) entered the regression equation, indicating that greater disease severity was associated with a stronger tendency to perceive Parkinson’s disease as a chronic, long-term condition. In the consequences dimension, younger age (*β* = −0.196, *p <* 0.05), lower educational level (*β* = −0.219, *p <* 0.05), and female gender (*β* = 0.184, *p <* 0.05) were associated with perceiving greater negative impact of the illness on life. In the personal control dimension, longer disease duration (*β* = −0.221, *p <* 0.05) was associated with lower perceived control, while older age (*β* = 0.202, *p <* 0.05) was associated with stronger perceived control. In the illness coherence dimension, female gender (*β* = 0.200, *p <* 0.05) was associated with a clearer, more coherent understanding of the illness. In the timeline cyclical dimension, lower educational level (*β* = −0.225, *p <* 0.05) was associated with a stronger belief that symptoms are cyclical and recurring. In the emotional representation dimension, female gender (*β* = 0.309, *p <* 0.01) and younger age (*β* = −0.249, *p <* 0.01) were associated with more severe negative emotional distress caused by the illness, indicating that young female patients experienced the most significant emotional distress. In conclusion, illness perception in patients with Parkinson’s disease is jointly regulated by objective clinical indicators and sociodemographic characteristics, with gender and age playing significant roles across multiple dimensions. Further details are shown in Table 4.

## 4 Discussion

### 4.1 Patients with Parkinson’s disease have significant negative illness perceptions

Illness perception refers to a patient’s understanding and awareness of their own illness, including their cognizance of symptoms and causes, with this perception having direct effects on their decisions regarding treatment, psychological state, rehabilitation, and self-management ability (23). Positive illness perception is critical for improving both treatment outcomes and quality of life. The current findings show that patients with PD have significant negative perceptions of their illness. Their scores on the CIPQ-R scale for the consequence 19.45 ± 3.03 and emotional representation 21.14 ± 3.44 factors were higher than those previously found in patients with stroke or diabetes (24, 25). This discrepancy may be attributable to the specific characteristics of PD, which is a progressive neurodegenerative disorder involving multiple systems. The gradually worsening burden of symptoms not only impairs patients’ daily life and functional ability but can also trigger negative emotions, such as anxiety, depression, and feelings of stigma (26). Therefore, improving illness perception in patients, especially their negative perception of the consequences of the disease, is crucial for optimizing treatment outcomes and enhancing quality of life.

### 4.2 Factors influencing illness perception in patients with Parkinson’s disease

The univariate analyses presented in Table 3 identified multiple factors associated with illness perception, including educational level, disease duration, and H-Y stage. Although unadjusted for confounders, these findings provide clinically relevant insights into at-risk populations. Notably, the patterns observed were broadly consistent with the multivariable regression results, reinforcing the robustness of these risk factors. The independent predictors identified by multiple linear regression are discussed in detail below.

#### 4.2.1 Factors associated with illness identity

The illness identity subscale reflects an individual’s awareness and cognitive integration of disease-related symptoms and the psychosocial impact of being labeled as having a chronic illness (27). This concept aligns with labeling theory, proposed by the sociologist Howard Becker, who posited that “once individuals are identified with a particular label, they tend to internalize and conform to the associated social identity (28)”. In the present study, women exhibited higher scores on the illness identity dimension than men, indicating greater sensitivity to and cognitive engagement with the illness label. This difference may result from gendered social expectations, as traditional norms often encourage men to be stoic and show emotional restraint, potentially leading to an underreporting or minimization of physical symptoms. In contrast, female patients may experience increased psychological distress due to the visible manifestations of PD, such as facial masking and limb tremors, and the perceived implications of these for social appearance and familial roles (29). Consistent with this, the present findings show that female patients also scored higher on emotional representation scores, suggesting a stronger tendency toward maladaptive emotional responses. The diagnosis of PD itself may intensify psychological burdens, prompting patients to attribute both somatic discomfort and emotional strain directly to the disease, thereby reinforcing a negative illness identity in a self-perpetuating cycle.

The Hoehn-Yahr (H-Y) staging scale, a well-established measure of motor symptom severity in PD, provides further clarification of this relationship. Higher H-Y stages are indicative of more advanced disease, and the findings showed a positive association between disease severity and illness coherence scores. As motor and non-motor impairments become more pronounced, patients frequently experience social stigma and interpersonal challenges, which, when combined with heightened self-awareness, can erode their self-esteem and distort their self-image. These factors collectively increase susceptibility to the labeling effect, reinforcing identification with a “sick role”.

Clinically, these findings underscore a need for targeted support for female patients and those with moderate to severe disease. Multidimensional interventions should be implemented to enhance quality of life. In terms of functional treatment, structured programs including facial muscle exercises and strategies for managing appearance, such as the use of warm-toned cosmetics to mitigate the social impact of facial masking, can help preserve the social confidence of patients. At the psychological level, cognitive behavioral therapy (CBT) is recommended to challenge beliefs about the illness, reduce over-attribution of symptoms to disease progression, and strengthen adaptive (30). Such approaches can effectively attenuate the negative consequences of illness labeling and enhance self-efficacy in disease management.

#### 4.2.2 Factors associated with timeline (acute/chronic)

The findings of this study indicate that marital status is associated with patients’ perceptions of the Timeline (acute/chronic). The Timeline (acute/chronic) reflects an individual’s belief about the expected duration of the disease. Patients who are widowed or unmarried tend to score higher on the chronicity dimension compared to married individuals, suggesting a stronger perception that their illness involves a prolonged course. Marriage functions as a critical source of social support, offering emotional understanding, practical resources, and spousal respect, which can strengthen patients’ self-confidence, alleviate psychological distress and social stress, and foster a more accurate understanding of the disease trajectory (31). Therefore, for patients who have lost their spouses, it is essential to strengthen family- and community-based support systems by encouraging active engagement with relatives, peer networks, and social organizations. Enhancing access to and utilization of social support, along with promoting patient support groups, can facilitate interpersonal communication, reinforce mutual encouragement, and cultivate a sense of companionship. Such interventions may help patients build resilience, improve psychological well-being, and enhance overall quality of life.

#### 4.2.3 Factors associated with consequence

The consequence dimension reflects the extent to which individuals believe that their disease will have significant negative consequences on their health and overall life trajectory. In this study, gender was identified as a key factor influencing consequence. Female patients reported higher levels of perceived consequence. This finding may be explained by several interrelated factors. First, sex-related differences are known to influence symptom expression and treatment responses in PD. As women generally have lower muscle mass, this may exacerbate the functional impact of disease-related rigidity and bradykinesia, resulting in more noticeable motor impairment (32). Second, differences in body composition and pharmacokinetics between women and men, such as disparities in drug absorption, distribution, and metabolism, may lead to suboptimal therapeutic outcomes if there is no individual tailoring of treatment regimens. Inadequate symptom control can also reinforce the perception of greater disease severity and rapid progression (32, 33).

These findings highlight the importance of targeted clinical interventions. For female patients, personalized rehabilitation programs, such as resistance training integrated with balance and coordination exercises, should be implemented to improve neuromuscular function and mitigate disability. Additionally, multidisciplinary teams should promote community-based educational initiatives and peer-led support sessions featuring resilient patient role models. The systematic integration of medical management, psychological counseling, and social support networks would enable healthcare providers to reframe illness perceptions, reduce the degree of catastrophic thinking about disease outcomes, enhance treatment adherence, and ultimately improve patient-reported outcomes.

#### 4.2.4 Factors associated with personal control

Personal control refers to an individual’s appraisal of their capacity to manage illness-related challenges, together with their effective utilization of available resources. The study findings showed that the perception of personal control declined significantly with prolonged disease duration. This trend is likely attributable to the progressive nature of PD, with overall worsening of both motor symptoms, such as rigidity and tremors, and non-motor manifestations, such as cognitive decline and depressive symptoms (34). The progressive accumulation of these impairments will compromise independence in the activities of daily living, undermining the patient’s confidence in managing the disease. Furthermore, prolonged treatment that does not achieve anticipated clinical outcomes may lead to feelings of disappointment and helplessness, while the continued financial burden incurred by medical care can restrict seeking of further treatments or supportive services, further eroding the sense of agency.

To address these challenges, healthcare providers should implement regular assessments of the functional status and symptoms of patients, enabling timely and individualized adjustments to pharmacological and rehabilitative therapies to maintain both the efficacy and relevance of treatment. The integration of evidence-based psychological approaches, such as mindfulness-based stress reduction (MBSR), can assist in the regulation of negative emotions, enhance emotional resilience, and improve coping strategies (35). Continuous long-term follow-up and dynamic monitoring are essential for the early identification and treatment of emerging complications. Moreover, the use of community-based support systems, engaging family caregivers, and facilitating peer support networks can strengthen the psychosocial resources of the patient, alleviate economic and emotional strain, and ultimately lead to a sense of control over their condition. Such comprehensive, patient-centered strategies are important for improving self-management capabilities and overall quality of life.

#### 4.2.5 Factors associated with timeline cyclical

Timeline cyclical refer to patient beliefs about the recurring and fluctuating nature of their illness. The findings of this study showed that individuals with lower educational levels scored higher on the timeline cyclical dimension, indicating a heightened perception of symptom recurrence that they were unable to control. These patients viewed the course of the disease as prolonged with little likelihood of significant improvement over time, leading to a more pessimistic outlook on treatment efficacy, consistent with the findings of Guo et al. (36).

To address this issue, healthcare providers should adopt tailored educational strategies for patients with limited formal education. The use of visual aids, such as diagrams, infographics, and instructional videos, as well as interactive teaching methods, can enhance comprehension and engagement. Health-related information should be presented in simplified, jargon-free language to ensure clarity and accessibility. Peer-led educational programs, particularly involving patients with similar educational backgrounds who had achieved stable symptom control, could serve as powerful tools to promote hope and reinforce confidence in treatment. Furthermore, treatment plans, including medication regimens and rehabilitation exercises, should be clear, practical, and easy to follow. Active involvement of family caregivers is essential to support adherence to treatment through consistent monitoring of medication intake and participation in rehabilitation activities.

The implementation of multilevel, patient-centered interventions would enable clinicians to assist patients with low levels of literacy in developing a more accurate understanding of disease fluctuations, mitigating maladaptive beliefs about chronicity and uncontrollability, and ultimately improving both treatment adherence and quality of life.

## 5 Conclusion

This study examined the current state of illness perception and its associated determinants in patients with PD. The findings revealed a general tendency toward negative illness representations. It was found that female patients, widowed individuals, those with longer disease duration, and those with lower educational levels were more likely to have negative perceptions of their condition. These results offer valuable insights for healthcare professionals for designing individualized interventions aimed at improving the psychological well-being and overall quality of life of PD patients.

## 6 Research limitations

Several limitations should be acknowledged. The study adopted a single-center, convenience sampling approach, which may limit the generalizability of the findings due to potential regional and selection biases. In addition, the cross-sectional design precludes causal inference, and some confounding factors, such as social support, were not fully controlled for. Furthermore, the uneven distributions of several categorical variables (e.g., marital status) resulted in extremely small subgroups in the ANOVA and regression analyses. Although these variables were excluded from the final multivariable regression models to avoid overfitting and biased estimates, the small subgroup sizes may have reduced the statistical power and increased the risk of Type I errors. Moreover, MDS-UPDRS scores were not included, and specific motor and non-motor symptoms were thus not fully captured. Finally, although family income was assessed as an exploratory sociodemographic variable, more direct factors, such as caregiver assistance and family support were not included in the analysis.

Future studies should adopt multi-center, longitudinal designs with larger and more diverse samples to verify the present findings and explore the dynamic changes in illness perception over time. Future research should also include MDS-UPDRS scores to provide a comprehensive assessment of specific motor and non-motor symptoms, as well as direct indicators, such as caregiver assistance and family support, to clarify their associations with illness perception.

## Data Availability

The raw data supporting the conclusions of this article will be made available by the authors, without undue reservation.

## References

[1] Zhu J Cui Y Zhang J Yan R Su D Zhao D . Temporal trends in the prevalence of parkinson's disease from 1980 to 2023: a systematic review and meta-analysis. Lancet Healthy Longev. (2024) 5:e464–79. doi: 10.1016/S2666-7568(24)00094-1, 38945129

[2] Jankovic J Tan EK. Parkinson's disease: etiopathogenesis and treatment. J Neurol Neurosurg Psychiatry. (2020) 91:795–808. doi: 10.1136/jnnp-2019-322338 32576618

[3] Li G Ma J Cui S He Y Xiao Q Liu J . Parkinson's disease in China: a forty-year growing track of bedside work. Transl Neurodegener. (2019) 8:22. doi: 10.1186/s40035-019-0162-z 31384434 PMC6668186

[4] GBD 2016 Neurology Collaborators. Global, regional, and national burden of neurological disorders, 1990-2016: a systematic analysis for the global burden of disease study 2016. Lancet Neurol. (2019) 18:459–80. doi: 10.1016/S1474-4422(18)30499-X, 30879893 PMC6459001

[5] Li M Ye X Huang Z Ye L Chen C. Global burden of Parkinson's disease from 1990 to 2021: a population-based study. BMJ Open. (2025) 15:e95610. doi: 10.1136/bmjopen-2024-095610, 40288800 PMC12035419

[6] Wal P Vig H Wal A Rathore S Pandey SS Jain NK . Role of nutraceuticals and physical activity in parkinson's disease risk and lifestyle management. Curr Aging Sci. (2023) 16:170–87. doi: 10.2174/1874609816666230515121717, 37638586

[7] Chaudhuri KR Azulay JP Odin P Lindvall S Domingos J Alobaidi A . Economic burden of parkinson's disease: a multinational, real-world, cost-of-illness study. Drugs Real World Outcomes. (2024) 11:1–11. doi: 10.1007/s40801-023-00410-1, 38193999 PMC10928026

[8] Hagger MS Orbell S. The common sense model of illness self-regulation: a conceptual review and proposed extended model. Health Psychol Rev. (2022) 16:347–77. doi: 10.1080/17437199.2021.1878050 33461402

[9] Morgan K Villiers-Tuthill A Barker M McGee H. The contribution of illness perception to psychological distress in heart failure patients. BMC Psychol. (2014) 2:50. doi: 10.1186/s40359-014-0050-3, 25520809 PMC4266484

[10] Hsiao C Chang C Chen C. An investigation on illness perception and adherence among hypertensive patients. Kaohsiung J Med Sci. (2012) 28:442–7. doi: 10.1016/j.kjms.2012.02.015, 22892166 PMC11916609

[11] Miglioretti M Mazzini L Oggioni GD Testa L Monaco F. Illness perceptions, mood and health-related quality of life in patients with amyotrophic lateral sclerosis. J Psychosom Res. (2008) 65:603–9. doi: 10.1016/j.jpsychores.2008.05.012, 19027451

[12] Arran N Craufurd D Simpson J. Illness perceptions, coping styles and psychological distress in adults with Huntington's disease. Psychol Health Med. (2014) 19:169–79. doi: 10.1080/13548506.2013.802355, 23767964

[13] Shinan-Altman S Werner P. Illness representations of dementia: a scoping review. Clin Interv Aging. (2019) 14:179–93. doi: 10.2147/CIA.S193316, 30697042 PMC6342146

[14] Eccles FJR Murray C Simpson J. Perceptions of cause and control in people with Parkinson's disease. Disabil Rehabil. (2011) 33:1409–20. doi: 10.3109/09638288.2010.533241, 21091132

[15] Evans D Norman P. Illness representations, coping and psychological adjustment to parkinson's disease. Psychol Health. (2009) 24:1181–96. doi: 10.1080/08870440802398188, 20204987

[16] Hurt CS Burn DJ Hindle J Samuel M Wilson K Brown RG. Thinking positively about chronic illness: an exploration of optimism, illness perceptions and well-being in patients with parkinson's disease. Br J Health Psychol. (2014) 19:363–79. doi: 10.1111/bjhp.12043, 23510498

[17] Meyer M Colnat-Coulbois S Frismand S Vidailhet P Llorca P Schwan R . Illness perceptions in pre-operative parkinson's disease patients. J Neural Transm (Vienna). (2023) 130:647–54. doi: 10.1007/s00702-023-02629-2, 37022502

[18] Leventhal H Phillips LA Burns E. The common-sense model of self-regulation (CSM): a dynamic framework for understanding illness self-management. J Behav Med. (2016) 39:935–46. doi: 10.1007/s10865-016-9782-2 27515801

[19] Postuma RB Berg D Stern M Poewe W Olanow CW Oertel W . MDS clinical diagnostic criteria for Parkinson's disease. Mov Disord. (2015) 30:1591–601. doi: 10.1002/mds.26424, 26474316

[20] Xu L Wang X Cui Y Tian Y Zhou N Zhang J . Illness perception characteristics and influencing factors in adult patients with myasthenia gravis in China. Brain Behav. (2022) 12:e2451. doi: 10.1002/brb3.2451, 34898040 PMC8785644

[21] EpiData Association. EpiData Software (Computer Software). Odense, Denmark: EpiData Association (2021).

[22] IBM Corp. IBM SPSS Statistics for Windows, Version 23.0 (Computer Software). Armonk, NY: IBM Corp (2015).

[23] Jia XL Sun J Chen J Chen HQ Lin BL Wang F . Research progress of illness perception assessment tools in chronic disease patients. Chin J Nurs. (2023) 58:1397–402. doi: 10.3761/j.issn.0254-1769.2023.11.018

[24] Jia GH. The impact of the three-stage health education model on disease knowledge cognition, medication adherence, and quality of life in patients with diabetes mellitus complicated by hypertension. J Qilu Nurs. (2018) 24:90–2. doi: 10.3969/j.issn.1006-7256.2018.11.036

[25] Ji HX Zhang JF Zhang L Wang K Gong GP Wang X. Analysis of the influencing factors on illness perception in patients with ischemic stroke. Chin J Arterioscler. (2016) 24:1001–6. doi: 10.20039/j.cnki.1007-3949.2016.10.006

[26] Peng N Li ZJ Liang J. Effect of nursing intervention based on interactive attainment theory on stigma, psychosocial adjustment and balance ability of patients with Parkinson disease. Med Innov China. (2024) 21:80–5. doi: 10.3969/j.issn.1674-4985.2024.22.019

[27] Rivera E Corte C DeVon HA Collins EG Steffen A. A systematic review of illness representation clusters in chronic conditions. Res Nurs Health. (2020) 43:241–54. doi: 10.1002/nur.22013, 32067248 PMC7186157

[28] Ahuvia IL Schleider JL Kneeland ET Moser JS Schroder HS. Depression self-labeling in U.S. college students: associations with perceived control and coping strategies. J Affect Disord. (2024) 351:202–10. doi: 10.1016/j.jad.2024.01.229, 38286232

[29] Wang YJ Liu WG Yu CY Yan L Zhang L Zhang WB . The characteristics and gender differences of non-motor symptoms in early diagnosed Parkinson’s disease. J Apoplexy Nerv Dis. (2022) 39:28–32. doi: 10.19845/j.cnki.zfysjjbzz.2022.0007

[30] Xiang L Wan H Zhu Y. Effects of cognitive behavioral therapy on resilience among adult cancer patients: a systematic review and meta-analysis. BMC Psychiatry. (2025) 25:204. doi: 10.1186/s12888-025-06628-3, 40050835 PMC11884023

[31] Yang C Zheng FQ. Investigation on social support and its influencing factors in patients with diabetic peripheral neuropathy. J Clin Nurs Pract. (2020) 6:92–6. doi: 10.11997/nitcwm.202005020

[32] Patel R Kompoliti K. Sex and gender differences in parkinson's disease. Neurol Clin. (2023) 41:371–9. doi: 10.1016/j.ncl.2022.12.001, 37030964

[33] Zucker I Prendergast BJ. Sex differences in pharmacokinetics. Handb Exp Pharmacol. (2023) 282:25–39. doi: 10.1007/164_2023_669, 37439843

[34] Shin JY Pohlig RT Habermann B. Self-reported symptoms of parkinson’s disease by sex and disease duration. West J Nurs Res. (2017) 39:1412–28. doi: 10.1177/0193945916670904, 27664144 PMC5505809

[35] Yu J Han M Miao F Hua D. Using mindfulness-based stress reduction to relieve loneliness, anxiety, and depression in cancer patients: a systematic review and meta-analysis. Medicine (Baltimore). (2023) 102:e34917. doi: 10.1097/MD.0000000000034917, 37713902 PMC10508374

[36] Guo FJ Xie MK Yan MX Yang QF. Status quo of self-management in young and middle-aged patients with chronic heart failure and its influencing factors. Nurs J Chin People's Liber Army. (2021) 38:5–08. doi: 10.3969/j.issn.1008-9993.2021.06.002

