# Supplementary material for: Factors influencing the perception of illness in patients with Parkinson’s disease
Source: Front Neurol. 2026 Apr 28;17:1773273. doi: 10.3389/fneur.2026.1773273 (PMC13160846; doi:10.3389/fneur.2026.1773273)
Supplement: Supplementary file 1 [file Supplementary_file_1.pdf]

Table 3. Comparative Analysis of Illness Perception Scores in Relation to Different Demographic Characteristics

Item	n	Illness identity	Timeline (acute/ chronic)	Consequence	Personal control	Treatment control	Illness coherence	Timeline cyclical	Emotional represent-tion
Age									
≤60	60	4.53±2.65	15.40±1.15	19.90±2.39	18.00±2.59	18.00±2.59	17.30±2.55	11.40±2.40	21.90±2.61
≥61	68	3.32±2.70	15.62±1.22	19.06±3.47	18.85±1.78	18.85±1.78	17.06±2.37	11.26±2.24	20.47±3.93
t-value		2.553	-1.033	1.611	-2.142*	0.1580	0.554	0.330	2.45*
Gender									
Male	70	3.37±1.82	15.60±0.94	18.91±3.15	18.63±2.35	19.66±2.13	16.74±2.49	11.09±2.08	20.29±3.82
Female	58	4.52±3.45	15.41±1.44	20.10±2.77	18.24±2.08	20.21±1.33	17.69±2.31	11.62±2.55	22.17±2.58
t-value		5.786*	0.775	5.033*	0.956	2.923	4.882*	1.714	10.260**
Marital status									
Married	124	3.90±2.74	15.45±1.50	19.40±3.02	18.48±2.21	19.90±1.85	17.15±2.49	11.32±2.33	21.11±3.49
Widowed or Unmarried	4	3.50±2.89	17.50±1.73	21.00±3.46	17.50±2.89	20.00±0.00	18.00±0.00	11.50±1.73	22.00±0.00
t-value		0.289	-0.357**	-1.037	0.868	-0.104	-3.831**	-0.151	-0.507
Educational level									
Primary school and below	70	4.37±3.07	15.43±1.37	19.97±2.28	18.57±2.55	20.06±1.42	17.63±2.42	11.71±2.38	21.63±2.86
Junior or senior high school	48	3.54±2.18	15.67±0.95	18.96±3.65	18.46±1.73	19.83±2.25	16.50±2.44	11.04±2.25	20.17±4.17
College degree or above	10	2.20±1.69	15.40±0.84	18.20±3.91	17.60±1.96	19.20±2.04	17.20±2.15	10.00±2.31	22.40±2.07

F-value		5.55*	0.617	2.16	0.989	0.90	3.12*	5.62*	3.384*
Family monthly income									
≤10000 RMB/month	56	3.93±2.87	15.36±1.05	19.25±3.28	18.64±2.45	19.93±1.93	17.18±2.43	11.25±2.07	20.71±3.70
1001-3000RMB/month	44	3.82±2.45	15.86±1.07	20.00±2.66	18.18±2.08	20.23±1.61	16.73±2.10	11.27±2.42	21.36±2.74
＞3000 RMB/month	28	3.93±2.97	15.29±1.51	19.00±3.06	18.50±2.03	19.91±1.83	17.86±2.45	11.57±2.63	21.64±3.86
F-value		0.023	3.22*	1.157	0.530	1.983	1.844	0.198	0.820
Type of Health Insurance									
medical insurance for urban employees	28	2.86±2.14	15.79±0.79	18.43±3.61	18.93±1.83	19.36±2.06	16.64±2.09	10.29±2.19	21.50±3.35
medical insurance for residents	12	3.00±0.85	15.00±1.21	18.67±2.67	17.50±2.32	20.67±1.16	16.83±3.33	11.50±2.30	21.50±0.52
New Rural Cooperative Medical Insurance Scheme	88	4.34±2.96	15.50±1.28	19.89±2.81	18.43±2.31	19.98±1.79	17.39±2.42	11.64±2.26	20.98±3.69
F-value		6.594**	2.460	2.991	1.757	2.430	1.107	3.840	0.766
Disease duration									
1-5 years	86	4.16±2.89	15.40±1.09	19.42±3.10	18.63±2.09	19.95±1.85	17.35±2.21	11.42±2.40	20.98±3.49
6-10 years	26	2.85±2.59	15.92±0.27	18.69±3.11	18.62±2.32	19.54±2.06	16.15±2.68	10.54±1.99	20.85±3.94
≥11 years	14	4.57±1.09	15.43±2.34	21.29±1.73	16.86±2.51	20.29±1.20	17.86±3.16	12.43±1.99	22.5±1.828
F-value		4.29*	8.42*	3.47*	4.07*	0.84	3.07	4.18*	1.39
H-Y staging									
Grade 1-2	64	3.34±2.81	15.31±1.30	19.16±2.86	18.31±2.10	19.78±1.95	17.19±2.81	11.38±2.44	21.03±3.49
Grade 2.5-5	64	4.44±2.56	15.72±1.05	19.75±3.19	18.59±2.36	20.03±1.70	17.16±2.05	11.28±2.19	21.25±3.41
t-value		-2.30*	-1.95	-1.11	-0.712	-0.774	0.072	0.029	-0.359

*p＜0.05，**p＜0.01
